# Edge Computing-Enabled Smart Agriculture: Technical Architectures, Practical Evolution, and Bottleneck Breakthroughs

**DOI:** 10.3390/s25175302

**Published:** 2025-08-26

**Authors:** Ran Gong, Hongyang Zhang, Gang Li, Jiamin He

**Affiliations:** School of Automotive and Traffic Engineering, Jiangsu University, Zhenjiang 212013, China

**Keywords:** edge computing, smart agriculture, real-time monitoring, agricultural equipment, agricultural vehicles

## Abstract

As the global digital transformation of agriculture accelerates, the widespread deployment of farming equipment has triggered an exponential surge in agricultural production data. Consequently, traditional cloud computing frameworks face critical challenges: communication latency in the field, the demand for low-power devices, and stringent real-time decision constraints. These bottlenecks collectively exacerbate bandwidth constraints, diminish response efficiency, and introduce data security vulnerabilities. In this context, edge computing offers a promising solution for smart agriculture. By provisioning computing resources to the network periphery and enabling localized processing at data sources adjacent to agricultural machinery, sensors, and crops, edge computing leverages low-latency responses, bandwidth optimization, and distributed computation capabilities. This paper provides a comprehensive survey of the research landscape in agricultural edge computing. We begin by defining its core concepts and highlighting its advantages over cloud computing. Subsequently, anchored in the “terminal sensing-edge intelligence-cloud coordination” architecture, we analyze technological evolution in edge sensing devices, lightweight intelligent algorithms, and cooperative communication mechanisms. Additionally, through precision farming, intelligent agricultural machinery control, and full-chain crop traceability, we demonstrate its efficacy in enhancing real-time agricultural decision-making. Finally, we identify adaptation challenges in complex environments and outline future directions for research and development in this field.

## 1. Introduction

Against the backdrop of a continuously growing global population and intensifying climate change, agricultural production—regardless of scale, from large-scale intensive farms to resource-constrained smallholder family farms—faces dual pressures to ensure both food security and sustainable resource utilization. Simultaneously, the digital transformation of agriculture, as a key countermeasure, is confronted with pervasive and multiple foundational constraints: field communication latency, low power consumption requirements of devices, and real-time decision-making bottlenecks form systemic challenges. These constraints are particularly pronounced in scenarios with relatively weak infrastructure, such as small farms, topographically complex regions, and mobile agricultural machinery operations. Within complex farmland environments, wireless signal propagation suffers significant degradation due to terrain-induced shadowing and crop canopy attenuation, severely compromising communication link stability. This exacerbates control command transmission delays, directly impairing agricultural machinery operational precision and reliability [1]. Concurrently, agricultural sensors deployed long-term in field environments lack stable power infrastructure, creating critical energy sustainability challenges. Limited battery longevity in sensor nodes restricts continuous monitoring of vital agricultural parameters [2]. More critically, agricultural equipment operating in dynamic scenarios demands millisecond-level control responsiveness. The inherent latency from data transmission and computational queuing in traditional cloud-based processing fails to meet the real-time decision-making time window requirements, resulting in delayed fault response and operational efficiency losses [3]. This triad of constraints reveals core bottlenecks in communication reliability, energy sustainability, and computational timeliness for agricultural digitization.

To address the aforementioned constraints, edge computing has emerged as a core enabling technology for agricultural digitalization by restructuring the data processing paradigm. As demonstrated in Table 1, its advantages are as follows.

(1) Low-latency response: Edge computing realizes distributed sinking of computing power through network topology reconfiguration, which essentially replaces the centralized processing paradigm of traditional cloud computing with physical spatial proximity, and establishes a real-time decision-making unit at the source of data collection. This architectural innovation completely circumvents the multi-hop routing delay of remote data transmission, so that the raw data stream captured by sensors is directly cleaned, features extracted, and decisions are generated at the edge nodes, significantly compressing the time overhead of the end-to-end processing link [4]. For instance, certain non-real-time yet computationally intensive analytical tasks—such as engine performance degradation prediction based on long-term time-series data and large-scale crop growth monitoring analysis using high-definition imagery—may be designed for execution in the cloud. However, if the generation of time-critical event detection or control commands that inherently require local rapid response—such as dynamic seed spacing compensation for seeders [5] and plant phenotypic feature extraction [6]—were to rely on cloud feedback, it would introduce response delays due to network latency and unpredictability. In agricultural vehicle dynamic control scenarios, this capability delivers millisecond-level responses for time-critical tasks, such as threshing drum blockage diagnosis in combine harvesters and real-time seeding spacing compensation in planters. It effectively addresses control command latency caused by network transmission fluctuations and computational queuing associated with traditional cloud processing paradigms. Moreover, the edge nodes further optimize the execution efficiency of complex algorithms (e.g., deep learning models [7]) through the parallel computing acceleration mechanism, so that computationally intensive tasks such as multi-sensor fusion sensing [8] and real-time path planning can still satisfy the strict time-window constraints under the resource-constrained environment [9]. This dual optimization based on spatial proximity and computational parallelism makes low-latency response a core enabling feature of edge computing to empower intelligent decision-making in agriculture, laying a technological cornerstone for precision operations in complex farmland environments [10].

(2) Bandwidth Optimization: Edge computing substantially decreases the volume of data requiring cloud transmission through raw data preprocessing, feature extraction, and data compression at network edge nodes [11]. In agricultural applications, this mechanism intelligently filters high-value decision features (e.g., crop stress indices, machinery fault signatures) while eliminating redundant raw data. This enables stable operation of bandwidth-intensive tasks—including UAV high-resolution image transmission and combine harvester multi-sensor monitoring—under constrained network conditions [12]. Furthermore, dynamic coordination in transmission protocols enhances bandwidth adaptability: edge nodes adjust data upload strategies based on real-time channel states, prioritizing transmission reliability for critical control commands during network congestion [13]. This three-layer optimization framework (“data reduction–feature refinement–dynamic adaptation”) provides inherent bandwidth optimization capabilities for large-scale agricultural IoT deployment.

(3) Data Localization: The decentralization of computing resources restructures data processing paradigms. Edge nodes process raw sensor data locally, eliminating the latency jitter and security risks associated with long-distance data transmission [14]. This architecture exhibits strong robustness against communication interruptions. Through pre-deployed lightweight models (e.g., micro neural networks), it maintains core decision-making capabilities in low-connectivity or offline scenarios, ensuring continuous operation for critical applications such as autonomous agricultural machinery navigation in hilly terrain and grid fault diagnosis during adverse weather events. Concurrently, minimizing network exposure of sensitive data substantially mitigates privacy leakage risks. When integrated with hardware-level encryption modules, the approach satisfies regulatory compliance requirements for highly sensitive information (e.g., healthcare data and industrial control commands). This spatial proximity-based processing mechanism constitutes a foundational enabler for high-real-time, high-privacy, and low-connectivity scenarios [15].

**Table 1:** Analysis of core advantages of edge computing in smart agriculture.

Advantage Dimension	Technical Mechanism	Agricultural Application Scenario	References
Low-Latency Response	Data source-end decision-making	Real-time adjustment of inter-plant distance in autonomous planters	[5]
	Distributed computing sink	Plant phenotypic feature extraction	[6]
	Parallel computing acceleration	Multi-sensor fusion perception in agricultural machinery	[10]
Bandwidth Optimization	Edge data filtering and feature extraction	High-definition image transmission by UAVs	[11,12]
	Dynamic transmission protocol adaptation	Agricultural machinery multi-sensor monitoring	[13]
Localized Data Processing	Local processing of raw data streams + lightweight model pre-deployment	Autonomous navigation of hillside orchards	[14]
	Minimization of network exposure	Protection of private data in farmland	[15]

The main contributions of this review are as follows:

1. We constructed a technical framework for edge computing-enabled smart agriculture, comprising a perception layer, a computation layer, and a communication layer.
2. We systematically reviewed the applications of edge computing in three core domains: precision crop management, intelligent agricultural machinery control, and whole-chain agricultural product traceability.
3. We conducted an in-depth analysis of critical challenges, including communication reliability, multi-source vibration coupling mechanisms, and multi-dimensional energy constraints. Furthermore, we proposed future research directions, such as optimization of data processing and intelligent decision-making, communication network enhancements, and breakthroughs in energy management and sustainability.

**Figure 1:**
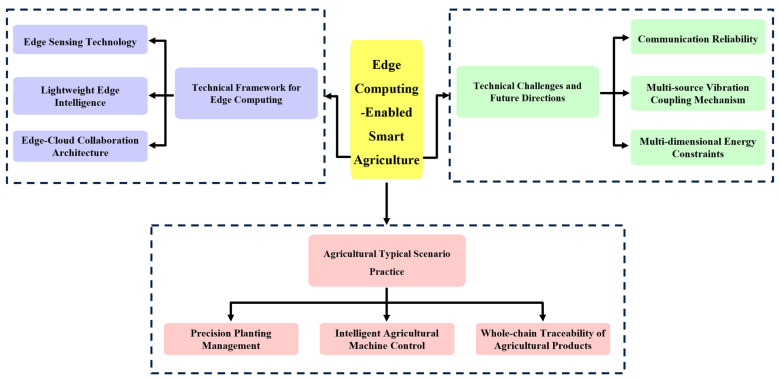
Edge computing-enabled smart agriculture.

The remainder of this paper is structured as follows. Section 2 introduces the fundamental technical architecture of edge computing: the sensing layer explores the innovative mechanism of agricultural-specific sensor design [16] (e.g., vibration-resistant sensing, low-power monitoring), the computing layer analyzes the optimization methods of lightweight model deployment and real-time decision engine, and the communication layer elaborates the edge–cloud cooperative architecture and the adaptation strategy of satellite-ground cooperative communication. Subsequently, Section 3, which focuses on the application layer, is dedicated to three core scenarios: precision farming [17] demonstrates implementation cases for crop phenotyping monitoring and intelligent irrigation systems; smart agricultural machinery examines solutions for autonomous navigation systems, operational quality optimization, and intelligent suspension control; and agricultural product traceability analyzes key technologies in harvest processing and quality safety management. Section 4 provides an in-depth analysis of technical bottlenecks: the technical bottlenecks focus on the core challenges of communication reliability, multi-source vibration coupling mechanism, and multi-dimensional energy constraints, which are revealed to be deeply rooted in the extreme dynamics and complexity of the agricultural production environment. Furthermore, it discusses prospective research directions. Finally, the study concludes by proposing pathways for cross-system collaborative optimization. The overall structure of this paper is depicted in Figure 1.

## 2. Technology Layer: Edge Computing Technology Architecture

**Figure 2:**
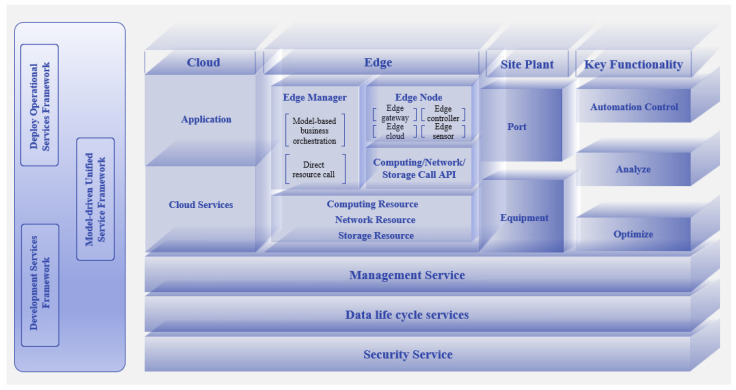
Hierarchical architecture of edge computing.

As shown in Figure 2, this study is grounded on a hierarchical edge computing architecture, which consists of three core logical layers.

(1) The cloud layer provides global scheduling;

(2) The edge layer supports real-time decision-making through managers and functional domain modules;

(3) The device layer connects physical terminals to form a closed loop.

This architecture provides two key supports for agricultural applications in this study: the edge-layer resource invocation interface ensures real-time response to agricultural conditions, and the horizontal service modules (e.g., Security Service) guarantee the trusted circulation of agricultural data. The specific agricultural scenario adaptation schemes and technical applications will be elaborated in the subsequent sections.

### 2.1. Edge Sensing Technology

#### 2.1.1. Crop Physiological Monitoring

Real-time analysis of crop physiological processes relies on the integrated application of multimodal sensing technologies. Spectral reflectance sensors enable non-destructive assessment of photosynthetic intensity by capturing the reflectance characteristics of the crop canopy in specific wavelengths and developing quantitative inversion models based on chlorophyll density and nitrogen accumulation [18]. Electrical impedance diagnostics use variable frequency excitation signals to penetrate plant tissues and analyze phase angle shifts in the complex impedance spectrum to reveal abnormal cell membrane potentials caused by potassium [19] or phosphorus [20] deficiencies, providing an electrophysiological basis for precise nutritional regulation. The transpiration monitoring sensor continuously tracks the water vapor diffusion flux through leaf stomata using a microfluidic humidity gradient capture device, and employs a canopy temperature compensation algorithm to eliminate solar radiation interference, establishing a dynamic evaluation system for water use efficiency [21].

#### 2.1.2. Environment Sensing Technology

The environment sensing layer of the agricultural edge computing system relies on multi-source heterogeneous sensing technologies to facilitate data transformation in the real world [22]. In complex farmland environments, multi-sensor fusion methods can significantly enhance the robustness of environmental sensing by processing heterogeneous data streams, such as spectra, vibrations, and images, in tandem [23]. Research has shown that such fusion frameworks can effectively leverage the complementary nature of multi-dimensional features to suppress noise interference, leading to a substantial improvement in classification accuracy for field-based environmental monitoring tasks [24]. The deployment of lightweight wireless sensor networks further extends the coverage area of edge sensing, and it has been shown that their tree topology reduces packet loss in field conditions [25]. Additionally, metal oxide semiconductor gas sensor arrays, optimized for localized signal processing, can enhance the accuracy of volatile organic compound (VOC) analysis during fermentation processes, achieving accuracy rates exceeding 94% [26]. Together, these technologies exhibit typical characteristics of edge sensing: Local preprocessing mechanisms (such as filtering and feature extraction) reduce uplink bandwidth pressure, dynamic power management strategies support long-term node operation, and protocol adaptive compatibility enhances the collaborative efficiency of heterogeneous devices. This technological framework provides critical infrastructure support for agricultural edge computing to address intermittent connectivity and energy constraints.

#### 2.1.3. Agricultural Product Quality Detection

In edge computing-driven smart agricultural systems, real-time in situ detection technologies for agricultural product quality are increasingly evolving towards miniaturization, low-power consumption, and high sensitivity. Among these, colorimetric sensor arrays (CSAs) mimic biological olfactory mechanisms and utilize selective reactions between specific chemical dyes and volatile organic compounds (VOCs) to produce a colorimetric response. For example, an array consisting of porphyrin and pH indicators can capture the aromatic characteristics of black tea [27]. When combined with a smart handheld device, this enables the geographic traceability of edible bird’s nests [28]. In grain storage monitoring, the technology allows for non-destructive, rapid assessment of wheat’s fatty acid content and the freshness of chicken meat (using TVB-N as an indicator), achieved through the optimization of feature dye combinations [29] and nonlinear regression algorithms [30]. One of its advantages is that no complex sample preprocessing is needed, and assay data can be processed directly at the edge node, significantly reducing cloud transmission load. The electrochemical aptamer sensor [31] leverages the aptamer’s high affinity for target molecules [32] and demonstrates excellent performance in trace pollutant detection. These sensors, enhanced with functionalized nanomaterials, enable ultra-sensitive detection of mycotoxins (e.g., zearalenone [33], aflatoxin [34]), pesticide residues (e.g., acetamiprid [35]), and illegal additives (e.g., rhodamine B [36]). However, current technologies still face challenges, including complex matrix interferences (e.g., the inhibition of electrochemical response by the wax layer on fruits and vegetables) and the influence of ambient temperature and humidity fluctuations on colorimetric stability. Future research should focus on developing interference-resistant designs to improve the robustness of edge-based detection models.

#### 2.1.4. Low-Power Design

The long-term reliable operation of agricultural edge sensing systems requires overcoming the energy constraint bottleneck [37]. Self-powered technology and dynamic dormant scheduling mechanisms offer innovative solutions to address this challenge. Self-powered sensors are generally implemented in two ways: the first involves developing environmental energy harvesting devices to power conventional sensors, while the second involves creating a new category of sensors—self-powered active sensors—that can generate electrical signals in response to stimuli from the surrounding environment [38]. For example, self-powered photoelectric sensors developed in previous studies enable continuous monitoring of chlorpyrifos residues in fruits and vegetables [39]. These systems typically integrate an energy storage unit capable of operating for several hours under low-light conditions. The sleep mode scheduling algorithm is based on an event-driven mechanism that automatically switches to a micro-amp standby state when no data collection tasks are present, thereby reducing the average power consumption of the wireless sensor network [40,41]. These two types of technologies form a “collection–storage–scheduling” energy-autonomous closed loop: vibration and light energy are efficiently converted and stored in micro-supercapacitors. The power management unit dynamically allocates power based on task priority, and the edge nodes wake the core circuits only during data sampling, enabling the farmland monitoring system to operate maintenance-free for up to a month. This energy-autonomous architecture provides essential hardware support for edge computing in remote agricultural environments.

#### 2.1.5. Novel Detection Technology

Within the framework of agricultural edge computing, innovations in novel detection technologies significantly enhance the sensing capabilities of edge nodes. Terahertz metamaterial sensors harness the electromagnetic resonance enhancement effect of artificial microstructures, enabling non-destructive analysis of the internal components of agricultural products in the 0.1–10 THz frequency band [42]. This mechanism depends on the modulation of terahertz waves by the dielectric properties of the target. As electromagnetic waves penetrate grain samples, differences in moisture content [43] and protein conformation may shift the characteristic absorption peaks. This change in frequency-domain response can be used to construct models predicting moisture distribution and mold levels [44]. Microfluidic chips condense lab-scale assays into centimeter-scale devices through the integration of microscale flow channels and functionalized electrodes. The multilayer paper-based microfluidic platform, combined with impedance time-series spectroscopy, enabled on-chip identification of organophosphorus pesticide species, achieving a classification accuracy of 93%. Its advantage lies in the reduction in reagent consumption to the microliter level and the elimination of immobilization steps, significantly decreasing detection cost and time, thereby making it compatible with the resource constraints of edge nodes [45].

### 2.2. Lightweight Edge Intelligence

#### 2.2.1. Model Compression and Acceleration

**Figure 3:**
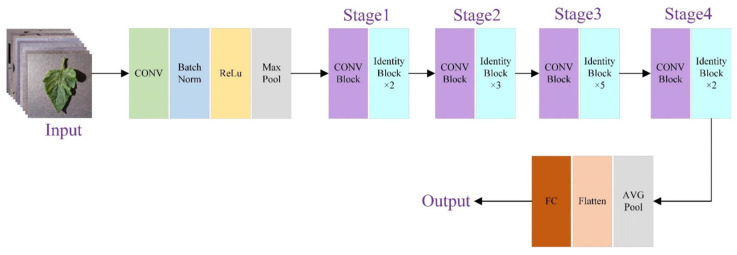
CNN architecture for agricultural leaf disease diagnosis [48].

The deployment of agricultural edge intelligence must overcome the dual challenges of computational power and energy consumption. Model compression and acceleration technologies, through algorithmic reconfiguration and hardware collaboration, enable efficient inference. At the algorithmic level, an attention-enhanced deep convolutional neural network (CNN) [46] has been employed for tomato leaf disease diagnosis [47], with its model architecture illustrated in Figure 3.

This model demonstrates clear advantages in terms of reduced network complexity and improved real-time performance. Furthermore, comparative experiments conducted on a public grape leaf disease dataset confirmed its superior recognition capability, achieving an average classification accuracy of 99.24% [48]. A multi-level fusion strategy of deep features proposes a fast and accurate detection method based on combining convolutional neural networks (CNNs) [49] with Support Vector Machines (SVMs). The SVM classifier trained using this method achieves classification performance comparable to the CNN model but in under 1 s, offering a significant advantage over the tens of minutes typically required to train CNN models [50]. Mainstream target detection frameworks, such as the YOLO series [51,52,53], are adapted for agricultural scenes using depthwise separable convolutions and channel pruning [54]. The structured pruning strategy removes redundant parameters or structures, compressing the model size and improving computational efficiency. At the hardware level, TensorRT optimizes inference through techniques like model quantization, reducing computation by lowering data type precision while maintaining high accuracy [55]. A complementary computation offloading strategy, along with model compression, creates an elastic computing architecture. When the edge node faces a complex task, the system decides whether to offload part of the computation based on real-time network bandwidth and server load [56]. The online collaborative data caching mechanism proposed in [57] reduces network transmission load and improves computational efficiency through real-time data sharing and multiplexing among distributed nodes in the edge computing environment. This mechanism is applicable to large-scale farmland monitoring and multi-agricultural machine collaboration scenarios in edge computing environments.

#### 2.2.2. Real-Time Decision Engine

(1) Adaptive Control: Real-time decision-making in agricultural edge scenarios requires control systems that are dynamically adaptable to the environment and optimized for resource constraints. Adaptive control strategies address farmland uncertainties by dynamically adjusting parameters. For example, sliding mode control demonstrates strong robustness in unmanned farm machine path tracking. According to [58], a fixed-time non-singular terminal sliding mode control scheme, based on adaptive perturbation observers, can stabilize lateral and heading deviations of unmanned farm tractors to an arbitrarily small neighborhood near the origin within a fixed time. Inverse step control compensates for deviations in crawler robot trajectories through real-time slip parameter estimation. This significantly improves localization accuracy and is particularly suitable for irregular terrains, such as hilly areas [59]. Both methods have been validated to complete status updates within millisecond-level cycles, meeting the real-time requirements of high-speed operations.

(2) Multi-agent Reinforcement Learning: Multi-agent Reinforcement Learning (MARL) enhances the decision-making system’s environmental adaptability. In task offloading, multiple agents collaborate to adapt to environmental changes and achieve load balancing in vehicle-mounted edge computing clusters. This approach outperforms the baseline in energy consumption, load state, and latency [60]. In resource scheduling, ref. [61] proposes a heterogeneous VEC supporting multiple communication technologies and task types. It includes an effective resource allocation strategy that meets ultra-reliable and low-latency communication (URLLC) requirements while minimizing system utility. Extensive simulation experiments demonstrate the effectiveness of this approach. The effectiveness of these methods arises from MARL’s quantitative modeling of group collaboration to achieve a near-global optimal allocation of edge resources.

### 2.3. Edge–Cloud Collaboration Architecture

#### 2.3.1. Terminal Layer

The terminal layer acts as the data collection front-end, significantly reducing edge transmission load through an embedded preprocessing mechanism. This layer uses lightweight computing units deployed in agricultural machines, drones, and environmental sensors to perform initial cleaning and feature extraction, effectively alleviating uplink bandwidth pressure [62]. For example, the nano-colorimetric sensors studied in [63] detect wheat mildew by extracting gas fingerprint features in a closed reaction chamber. The original data is significantly compressed, and only classification results are transmitted to the edge node. This preprocessing typically limits computation delay to milliseconds, with energy consumption accounting for only 8–15% of the total power usage, demonstrating the energy efficiency of lightweight computation at the terminal layer.

#### 2.3.2. Edge Layer

The edge layer functions as an intermediate computing layer between terminal devices and cloud data centers, offering near-end data processing and real-time decision-making through geographically distributed nodes. This tier leverages computing resources in farmland edge servers, agricultural machinery on-board computers [64], and other facilities to perform medium-complexity analysis tasks near the data generation source, alleviating latency and bandwidth pressures associated with cloud-based processing [65]. Its core value lies in enabling low-latency responses for time-sensitive agricultural operations. For example, the grain loss monitoring system of combine harvesters [66] achieves millisecond-level decision latency by processing piezoelectric sensor signals in real time through on-board edge units, representing a significant acceleration compared to traditional cloud-based processing. Similarly, the stereoscopic vision navigation module [67] relies on lightweight models deployed at the edge layer to perform crop boundary detection, supporting autonomous steering control of agricultural machinery at a rate of 49 ms per frame. The edge layer also undertakes dynamic resource scheduling [68], elastically reallocating tasks based on network conditions and computational load—for instance, assigning soil moisture analysis to nearby idle nodes to accelerate irrigation decision-making. This layer continuously receives model optimization parameters delivered from the cloud via an incremental learning mechanism, forming a collaborative “edge execution–cloud evolution” closed loop. While ensuring the real-time performance of farming operations, it provides a computing paradigm that offers both adaptability and scalability for complex agricultural environments.

#### 2.3.3. Cloud

The cloud layer serves as the global intelligent hub, responsible for core functions including long-term data analytics and complex model optimization [69]. Leveraging high-performance computing resources, this tier integrates multi-source heterogeneous agricultural data to construct macro-scale decision-making-oriented deep computational models. Cloud-based training processes typically focus on cross-growth-cycle time-series analysis tasks. For instance, joint modeling incorporating multi-seasonal field environmental parameters and crop phenotypic data can reveal underlying correlation patterns between temperature gradients, precipitation regimes, and yield fluctuations. Such models significantly surpass edge-layer localized models in both parameter scale and training complexity, deriving their value from capturing the long-range dependencies arising from nonlinear interactions within agricultural systems. The cloud and edge layers establish a functionally complementary, synergistic paradigm [70]: the edge layer prioritizes millisecond-response real-time control tasks, while the cloud layer is dedicated to uncovering agricultural operational patterns across spatiotemporal scales. This layered architecture maximizes the cloud’s strengths in deep modeling while ensuring system responsiveness through the edge layer’s agile execution, thereby establishing a resilient computing infrastructure for precision agriculture that simultaneously addresses macro-scale optimization and micro-scale precision.

#### 2.3.4. Communication Protocol Optimization

Communication protocol optimization plays a pivotal role in ensuring efficient and reliable operations within edge–cloud collaborative architectures. In smart agricultural systems, where edge nodes (including field-deployed soil sensors, weather stations, pest monitors, and agricultural machinery controllers) are typically situated in remote, resource-constrained environments with dynamic network conditions, conventional communication protocols designed for stable data center networks often fail to meet the core requirements of low latency, high throughput, and minimal overhead essential for real-time field monitoring and precision farming operations [71]. Therefore, the optimization of communication protocols for agricultural edge–cloud collaboration scenarios has become a major focus in both research and practice. Key optimization approaches involve streamlining protocol stacks to reduce processing overhead while enhancing adaptability to common agricultural network fluctuations, including bandwidth variations, latency jitter, and intermittent connectivity. Current research emphasizes protocol header compression, lightweight improvements to connection establishment and maintenance mechanisms, targeted tuning of congestion control algorithms (to prevent critical command loss in bandwidth-constrained farm networks), and scenario-specific redesign or customization of transport-layer protocols [72]. Furthermore, given the heterogeneous nature of data transmitted between agricultural edge devices and cloud platforms (as presented in Table 2), protocol design must holistically consider data prioritization, real-time transmission requirements, and agriculture-specific security and privacy constraints to develop differentiated quality-of-service transmission strategies. Emerging technologies such as semantic communication [73] are being investigated to reduce redundant data transmission and improve information delivery efficiency in specific applications. The continuous refinement of communication protocols is crucial for fully realizing the potential of agricultural edge–cloud collaboration, particularly in ensuring end-to-end service quality for precision agriculture, with special significance for latency-sensitive applications operating in bandwidth-constrained environments.

In summary, the technical architecture of edge computing-empowered smart agriculture spans multiple layers, from perception and intelligence to collaboration. Each core technology offers distinct advantages suited to specific agricultural scenarios, while simultaneously facing challenges stemming from the complexity of agricultural environments. The correspondences, core strengths, key challenges, and typical application scenarios of these technologies are summarized in Table 3.

## 3. Application Layer: Agricultural Typical Scenario Practice

### 3.1. Precision Planting Management

#### 3.1.1. Crop Phenotype Monitoring

**Figure 4:**
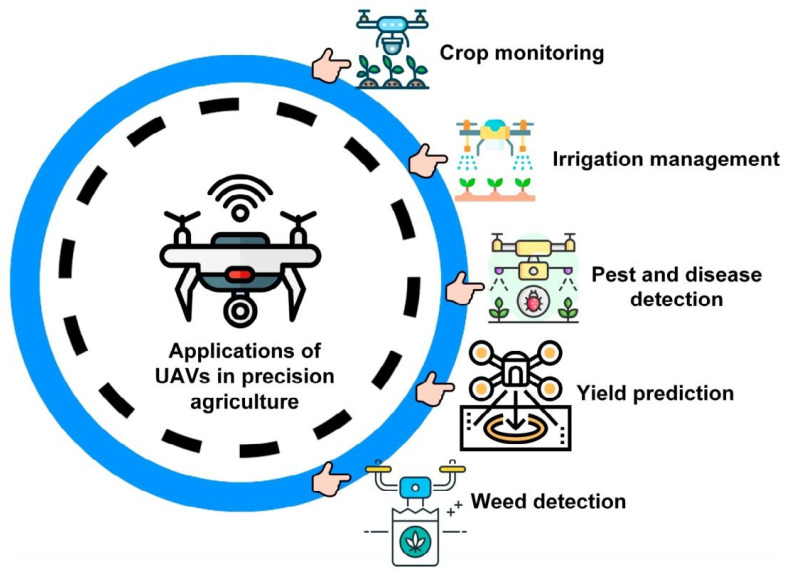
Applications of unmanned aerial vehicles (UAVs) in precision agriculture [77].

In the field of crop phenotyping monitoring, unmanned aerial vehicle (UAV) platforms have become deeply aligned with the core themes of precision agriculture due to their high mobility and flexibility, effectively addressing challenges such as dynamic canopy structural variations and environmental heterogeneity. As illustrated in Figure 4, key applications across crop monitoring, irrigation management, yield prediction, and weed detection visually reflect the emerging research trend positioning UAVs as a core platform in air–ground collaborative sensing systems [74,75]. Equipped with multispectral sensors [76], UAVs efficiently capture spectral reflectance and thermal infrared features of the crop canopy [77]. These raw data are processed in real-time by edge nodes to quickly extract information, such as vegetation index and chlorophyll fluorescence parameters. This provides minute-level data support for analyzing the crop’s physiological state, reduces cloud transmission delays, and enhances efficiency in large-scale farmland phenotype monitoring. These real-time phenotypic parameters can be used in elemental content prediction models (e.g., nitrogen and phosphorus) [78] and linked with ground-based [79] electrophysiological monitoring equipment to form a multi-scale sensing network. For example, the edge computing node combines wide-area UAV scanning data with high-frequency ground sensor samples to create a crop water stress response model, providing real-time crop water demand information and directly controlling the variable irrigation system for precise water replenishment [80].

#### 3.1.2. Pest and Disease Prevention and Control

**Figure 5:**
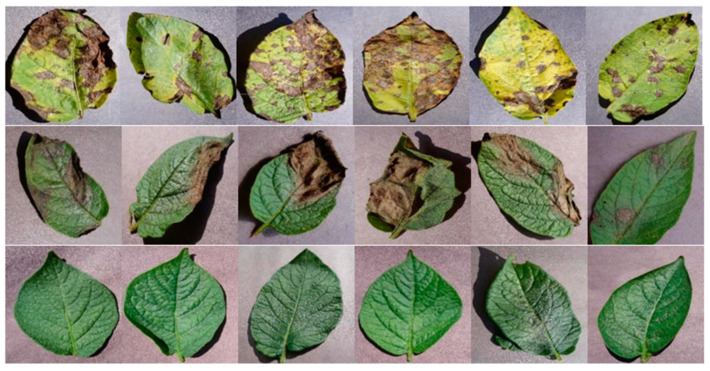
Example of potato leaf diseases. Three types of potato leaves including early blight, late blight, and healthy [84].

In precision planting management, early and accurate disease prevention and control are critical to safeguarding agricultural output. With the advancement of deep learning [81], computer vision-based leaf disease diagnosis systems are replacing traditional, experience-based visual inspection methods [82], providing data-driven decision support for field disease management [83]. For example, in potato cultivation, early and late blight diseases (as shown in Figure 5) [84] can be diagnosed using a lightweight MobileNet convolutional neural network. The system automatically extracts and classifies disease spot features in complex field backgrounds, achieving an accuracy of 96.6%, which significantly reduces subjective bias in manual diagnoses and offers a scalable, efficient solution for early disease identification and mitigation [85]. Similarly, in cotton leaf disease diagnosis, a transfer learning mechanism optimizes the feature extraction of pre-trained models, enabling effective differentiation between anthracnose and blight under varying environmental conditions (e.g., high humidity, dusty environments). The system maintains high recall even for translucent, water-soaked spots at the early stages of disease development [86].

### 3.2. Intelligent Agricultural Machine Control

#### 3.2.1. Autopilot Systems

In the intelligent evolution of agricultural machinery, breakthrough progress in autonomous navigation systems has been achieved through deep integration of multimodal perception and edge-based decision-making [87]. To address the inherent limitations of satellite navigation in row-cropping scenarios (e.g., weak recognition of ridge structures, sensitivity to signal obstruction) [88,89], tightly coupled vision-laser-inertial guidance technology has emerged as a mainstream solution. Taking combine harvesters as an example, a tightly coupled optimization engine fuses GNSS raw data, visual semantic features, and laser point clouds to generate centimeter-level global pose estimates [90]. Field validation demonstrates that this approach controls the average lateral deviation within 3.8 cm during inter-row operation, achieving a 42% improvement over traditional monocular vision, while maintaining a positioning error below 5 cm even after 30 s of signal loss [91]. To mitigate navigation reliability degradation caused by crop occlusion, a semantic map diffusion model based on a noise iterative mechanism effectively reconstructs ridge structures, significantly outperforming adaptive threshold segmentation and conventional contour detection algorithms in occlusion scenarios [92].

**Figure 6:**
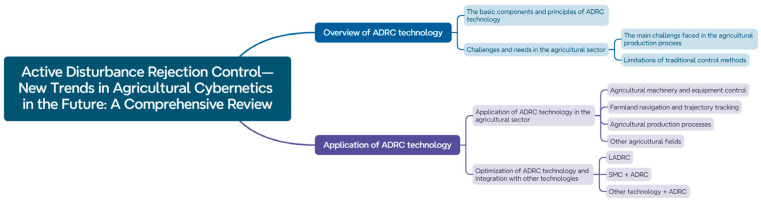
Main application categories of ADRC technology in agriculture [93].

Active Disturbance Rejection Control (ADRC) is concurrently introduced: through an extended state observer, it estimates total system disturbances (such as terrain variation and mechanical vibration) in real time and dynamically compensates for interference via a nonlinear feedback control law, substantially improving trajectory tracking accuracy under complex operating conditions. Its main application categories in agriculture are shown in Figure 6, covering fundamental principles, challenges in agricultural scenario adaptation, and innovations in multi-domain applications [93]. With the maturation of high-precision navigation technologies, agricultural machinery operation is transitioning from single-unit automation [94] toward group intelligence and collaboration [95]. Multi-machine systems achieve centimeter-level cooperative control through dynamic formation strategies: agricultural machinery is grouped dynamically based on field size and terrain parameters, with an optimization function modeled for each group that incorporates operational sequence, harvesting radius, and velocity coordination. This collaborative mechanism is further integrated with supply chain data, forming a closed-loop “operation-storage-transport” system that significantly enhances resource allocation efficiency [96]. An end-to-edge decision-making system ensures real-time performance of critical control tasks: the edge computing unit supports millisecond-level response for applications such as seeding spacing compensation and threshing drum blockage diagnosis [97]; dynamic route planning based on maturity analysis greatly reduces non-working travel distance; and optimization of the ADRC execution layer improves the stability of hydraulic steering systems under sudden load changes, yielding a notable increase in response speed compared to traditional hydraulic control.

#### 3.2.2. Operation Quality Optimization

Operation quality optimization is a key aspect of improving the effectiveness of intelligent agricultural machinery systems, and significant breakthroughs are being made through the synergy of sensing technology and edge decision-making. In the combine harvester operation scenario, the grain loss monitoring system uses piezoelectric sensor arrays [98] combined with a discrete element method collision model [99] to monitor the distribution dynamics of grains and impurities at the end of the cleaning sieve in real-time. Field test results demonstrate that the measurement error recorded by the sensors is less than 4.48% when compared to manual measurements. By utilizing these real-time loss data, a tailings grain loss distribution function and a monitoring mathematical model incorporating relevant variables [100] are integrated with the yield distribution mapping system [101], thereby constructing a spatiotemporal “loss-yield” correlation model. This model provides a quantitative basis for the dynamic adjustment of harvesting parameters. Compared to real-time harvesting monitoring, tillage quality optimization focuses more on predictive control before operation. To address plowing depth fluctuations, a plowing depth detection and control method based on attitude estimation and online calibration of model parameters achieves stable control of plowing depth through the online calibration of plow body pitch angle parameters [102]. In tillage resistance prediction [103], a discrete element simulation and fuzzy logic model [104] constructs an analytical framework for the soil-machine interaction relationship. Field validation demonstrates that the model’s prediction accuracy for lateral force and vertical force reaches correlation coefficients of 0.95 and 0.84, respectively.

#### 3.2.3. Intelligent Suspension Control

**Figure 7:**
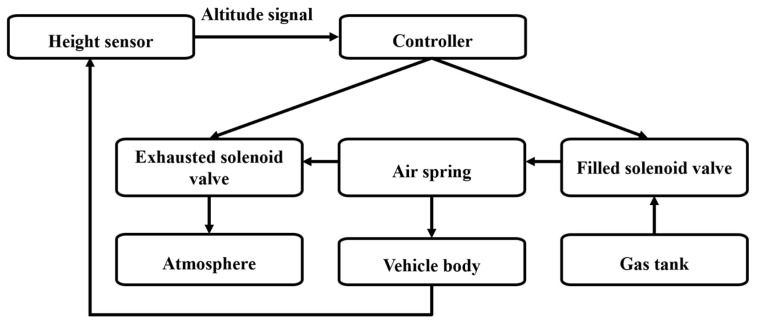
Schematic diagram of the inflation/deflation circuit in the electronically controlled air suspension system.

Building upon centimeter-level navigation in autonomous systems and the established “loss-yield” spatiotemporal model for operational quality optimization, dynamic suspension regulation emerges as the critical nexus bridging these components. In agricultural applications, traditional passive suspensions—constrained by mechanical limitations—struggle to address coupled low-frequency terrain undulations and high-frequency vibrations characteristic of complex field environments. Edge-based active control systems overcome this bottleneck through distributed computing nodes that process high-velocity sensor data streams. For instance, boom sprayers equipped with electro-hydraulic active suspensions deploy DSP controllers at the edge layer, substantially reducing closed-loop cycles for signal acquisition, multi-sensor fusion, and servo valve control while mitigating communication latency inherent to centralized architectures. Concurrently, velocity feedforward PID control (VFPID) implemented on edge nodes enhances the boom’s adaptive terrain-following capability, significantly improving pesticide deposition uniformity [105]. Similarly, Figure 7 demonstrates an edge control system for agricultural transport vehicles employing electro-pneumatic suspensions with adaptive PID algorithms. This system locally adjusts vehicle height through real-time fusion of vehicle dynamics and road condition data at edge nodes, minimizing oscillation during height transitions and substantially improving transport stability and ride comfort [106]. The core innovation lies in offloading decision logic to proximity with physical actuators, where operational condition-adaptive mechanisms markedly improve system resilience in complex farmland environments. As edge computing and cloud platforms achieve deeper integration, suspension control strategies may evolve toward higher-level autonomous decision paradigms.

### 3.3. Whole-Chain Traceability of Agricultural Products

As shown in Table 4, the whole-chain traceability system for agricultural products achieves breakthroughs in key stages through technological innovations.

#### 3.3.1. Harvesting and Processing

As a core component for ensuring food safety and enhancing value-chain transparency, whole-chain traceability of agricultural products relies on the precise and efficient monitoring of critical quality parameters from the point of harvest to the processing terminal. In the harvesting stage, non-destructive and rapid quality grading technologies are widely adopted to ensure the quality of raw materials. Among them, near-infrared spectroscopy [107,108], due to its non-destructive and high-efficiency characteristics, has become the preferred method for the online grading of internal fruit quality attributes such as sugar content, acidity, and firmness, thereby providing standardized raw materials for subsequent processing [115]. Vibrational spectroscopy offers a complementary analytical approach by capturing the response signals of fruits under mechanical excitation, effectively correlating key indicators such as hardness, soluble solids content, and even sensory quality, thus providing a novel technological pathway for assessing the ripeness and quality of fruits such as kiwifruit [116]. Furthermore, in the stage of agricultural product processing, particularly in areas involving biological conversion processes, real-time online monitoring technologies are crucial for ensuring process stability and product consistency. Fermentation, as a common biochemical reaction in agricultural processing, requires precise control of core parameters (e.g., key metabolite concentrations, degree of fermentation), which directly determine the flavor and quality of the final product. Studies have demonstrated that monitoring systems based on spectroscopic principles (e.g., NIR spectroscopy, visible–NIR spectroscopy) combined with advanced chemometric models have been successfully applied to real-time prediction of quality indicators such as catechin content and the tea polyphenol/amino acid ratio during black tea fermentation [109], as well as the online identification of dynamic changes in volatile organic compounds during the oxidation stage of oolong tea [110]. Similarly, in traditional brewing processes, portable spectroscopic analysis systems have been proven effective in simultaneously tracking real-time changes in total sugar, alcohol content, and pH during yellow wine fermentation, significantly improving process controllability and product standardization [117]. By enabling continuous, non-invasive monitoring of key processing parameters, these technologies provide robust technical support for optimizing processing parameters, ensuring product quality consistency, and realizing intelligent production control [118].

#### 3.3.2. Quality and Safety

As the core objective of the full-chain traceability system, agricultural product quality and safety rely on the precise monitoring of key risk factors, among which efficient monitoring of mycotoxins and pesticide residues constitutes a critical link in risk prevention and control. In the field of mycotoxin detection, innovations in aptamer sensing technology have significantly improved field detection capabilities. For example, the self-enhanced electrochemiluminescence sensor for zearalenone (ZEN) enables a wide linear detection range from 10 fg/mL to 10 ng/mL in maize flour [33]. The ratiometric fluorescence strategy optimizes the detection limit of ZEN to 0.32 pg/mL through the regulation of internal filtering effect [119], while the photo-induced electron transfer mechanism further improves sensitivity to 0.1 pg/mL [120]. For trace analysis of ochratoxin A, the synergistic effect of dendrimeric magnetic nanocomposite substrates and functionalized gold probes significantly enhances sensitivity through surface-enhanced Raman scattering (SERS) [111]. In contrast to mycotoxins, rapid identification of pesticide residues has also made significant progress. The rapid identification of pesticide residues is evolving from single-indicator screening to multi-target integration. Enzyme inhibition-regulated fluorescence sensing has made breakthroughs, optimizing the detection limit of organophosphorus pesticides to 0.05 ng/mL through a synergistic “off-on-off” detection mechanism based on up-converted nanoparticles and copper ions [121]. The innovative application of fluorescence resonance energy transfer (FRET) technology has enabled the simultaneous detection of paraquat (0.18 ng/mL) and carbendazim (0.45 ng/mL) through black phosphorus nanosheets (BPNSs) [112], significantly enhancing detection efficiency in complex matrices. Furthermore, engineered SERS substrates improve field applicability. Mesoporous silica-loaded ordered gold nanoparticles show excellent stability (relative standard deviation ≈ 3%) [122], while signal-optimized floral silver nanostructures (AgNPs), combined with solid-phase extraction, enable highly sensitive detection of pesticides like methomyl in tea at levels as low as 10^−4^ μg/mL [113]. It is noteworthy that the inner filter effect-based sensing utilizes the dithizone–cadmium complex (DZ–Cd^2+^) system [123], in synergy with the enzyme inhibition-oxidation triggering mechanism [124], creates a multi-modal pesticide residue recognition network. These technological advancements have been ultimately implemented through a microfluidic-integrated platform for scenario-specific adaptation [114]. By integrating optical and electrochemical sensing modules, this platform significantly enhances the efficiency of on-site detection. Current progress is driving a paradigm shift in pesticide residue monitoring from laboratory-based analysis to real-time, in-field detection, thereby providing dynamic assurance for whole-chain traceability of agricultural products.

## 4. Challenges and Future Directions

Edge computing-based smart agriculture faces systemic bottlenecks arising from the dynamic and complex nature of agricultural production environments. Breakthroughs in this area rely on cross-domain collaborative innovation and deeper integration of emerging technologies.

### 4.1. Challenges and Particularities

(1) Communication Reliability Bottlenecks: Field deployment confronts issues such as network dead zones, terrain-induced signal obstruction [125], and fluctuations in dielectric constant caused by dynamic soil moisture variations [126,127]. These are further compounded by multipath wireless signal scattering due to machinery vibration [128], significantly increasing the packet loss rate during the transmission of critical data such as grain flow [129] and detailed canopy structural parameters [130,131]. This severely limits the application of advanced sensing technologies that depend on high bandwidth and low latency—including visual 3D modeling [132,133,134] and ultrasonic time-series analysis—thereby degrading the accuracy of crop growth monitoring and precision management.

(2) Multi-Source Vibration Coupling Mechanisms: Time-varying loads and multi-source coupled vibrations induced by unstructured field operations—such as combined impact in balers with combustion excitation [135] and header vibration in harvesters [136]—exhibit nonlinear behaviors far exceeding those in industrial settings. These directly threaten the structural integrity of critical mechanical and electrical components (e.g., differential gearboxes [137] and in-wheel motor systems [138,139,140]), and considerably impair operational quality (e.g., crushing rate [141] and seeding depth uniformity [142]). The unique vibration transmission paths excited by mobile platforms and uneven terrain constitute a core challenge for suspension system design and reliability assessment.

(3) Multi-Dimensional Energy Constraints:

a. Unstable Energy Supply: Remote farmland often depends on renewable energy sources [143]. Cloudy and windless conditions can easily trigger node dormancy, interrupting high-dynamic tasks such as the “perception–decision–execution” closed loop in smart irrigation [144,145,146,147]. This leads to model inaccuracy, water–fertilizer imbalance, and undermines confidence in technology adoption [148,149,150].

b. Concentration of High Energy-Consumption Scenarios: Mobile edge devices are required to support computationally intensive tasks [151] and high-power consumption actuators (e.g., irrigation valves [152,153], curtain motors [154], and hydraulic systems [155]). Traditional batteries struggle to meet sustained operational demands, and voltage sags threaten system reliability.

c. Energy-Computing Trade-off: Among various energy constraints, the energy-computing coordination conflict between mobile smart terminals and edge computing nodes is particularly prominent [156]. Under weak network coverage, offloading computational tasks to meet low-latency requirements [157] forces terminals to increase transmission power, surging communication energy consumption, which may offset the benefits of offloading. Concurrent task offloading from multiple devices [158] causes congestion and queue accumulation, increasing both overall system energy consumption and latency [159]. Achieving a fine-grained balance among terminal energy consumption, communication energy consumption, edge energy consumption, and latency is exceptionally challenging [160], and dedicated energy-saving techniques are still in the exploratory stage.

In summary, the core challenges faced by agricultural edge computing during deployment (as shown in Table 5) exhibit significant environmental coupling characteristics. On one hand, the coupling of terrain obstruction and dynamic variations in soil dielectric properties leads to a notable increase in communication packet loss rates, severely degrading the accuracy of crop canopy modeling and forming a bottleneck for precise perception. Simultaneously, heterogeneous vibrations excited by unstructured field environments directly disrupt the uniformity of seeding operations, impairing the reliability of the execution process. More critically, the fragility of the energy chain not only risks interrupting irrigation decision-making but also, when combined with the high energy consumption required for computational offloading in weak network environments, imposes rigid constraints on the “perception–decision–execution” closed loop. These systemic bottlenecks, stemming from the unique dynamic characteristics of agricultural production environments, urgently require breakthroughs through cross-domain collaborative innovation.

### 4.2. Future Evolution Pathways

To advance smart agriculture beyond current environmental constraints and performance bottlenecks, edge computing technology requires systematic and collaborative innovation across key dimensions, including architecture, decision-making, energy, communication, and security. Its evolution will no longer focus solely on improving individual technical metrics but will strive to construct an efficient, reliable, and sustainable integrated solution. The future development pathways primarily revolve around the following five core dimensions:

(1) Evolution of Intelligent System Architecture: Distributed “intelligent edge” nodes with enhanced processing capabilities will enable real-time responsiveness to environmental dynamics (e.g., weather, soil, crops) and support personalized management. Efficient edge–cloud collaboration—where the edge handles real-time decision-making and the cloud performs in-depth analysis—will optimize computational resource allocation.

(2) Optimization of Data Processing and Intelligent Decision-Making: Edge computing, integrated with artificial intelligence and machine learning [161,162], facilitates localized real-time analysis of perceptual data and powers intelligent decision engines (e.g., for precision irrigation). This supports the automation and coordinated operation of agricultural machinery, driving the transition toward intelligent production.

(3) Breakthroughs in Energy Management and Sustainability: Advancements in self-sustaining energy technologies—such as integrated energy harvesting—and intelligent power management strategies will maximize the efficiency of renewable energy sources like solar-wind systems. These innovations ensure continuous system operation even in low-power environments.

(4) Communication Network Optimization and Expansion: Customized agricultural communication protocols will ensure stable and secure data transmission under weak or interrupted network conditions. Technologies such as 6G and low-earth orbit (LEO) satellites will enhance low-latency, high-throughput service capabilities across wide-area coverage, enabling remote monitoring and processing.

(5) Enhanced Data Security and Privacy: By incorporating encryption algorithms and blockchain technology, edge computing can ensure secure local data storage, protect sensitive information, and guarantee transmission integrity. This strengthens support for agricultural product traceability and supply chain management.

## 5. Conclusions

Edge computing technology is emerging as a core engine driving the transition of smart agriculture toward real-time and intelligent operations. By deploying computing capabilities closer to the network edge, it effectively addresses critical issues such as massive data processing, low-latency response, and energy constraints in agricultural production. This paper systematically elaborates on the technical architecture, typical applications, and challenges of edge computing in agriculture, highlighting its significant potential to enhance perceptual accuracy, decision-making efficiency, and operational reliability.

Currently, the implementation of edge computing in complex agricultural environments is hindered by three primary challenges.

(1) Decreased communication reliability due to terrain obstruction, dynamic fluctuations in soil dielectric properties, and machinery vibration;

(2) Severe impacts on the structural integrity of agricultural machinery components and operational quality caused by multi-source vibration coupling excited in unstructured field environments;

(3) Multi-dimensional energy constraints arising from the instability of renewable energy supply, the concentration of high energy-consumption tasks, and the energy-computing coordination conflict.

These challenges deeply reflect the unique dynamics and complexity of agricultural environments, imposing higher requirements on the adaptability, robustness, and energy efficiency of edge computing systems.

In the future, the evolution of intelligent edge architectures, breakthroughs in lightweight artificial intelligence algorithms, the integration of satellite–terrestrial collaborative communication technologies, and innovations in dynamic energy management strategies will collectively propel agricultural edge computing toward higher efficiency, reliability, and sustainability. The deep integration of edge computing with end-to-end agricultural management will accelerate the fundamental transformation of agricultural production modes from precision-based to autonomous and green paradigms. Ultimately, this will provide key technological support for ensuring global food security, optimizing resource utilization, and promoting environmental sustainability.

This paper offers a systematic reference for theoretical and applied research on edge computing in smart agriculture. Subsequent efforts should focus on the collaborative optimization of multi-physical field coupling mechanisms involving communication, vibration, and energy, the co-design of edge-specific hardware, algorithms, and protocol stacks, as well as the development of cross-platform standardized frameworks. These initiatives will facilitate the transition of edge computing from technological validation to large-scale agricultural deployment, empowering resilient, efficient, and sustainable modern agricultural systems.

## Figures and Tables

**Table 2:** Data types and their transmission characteristics.

Data Type	Typical Application Scenario	Real-Time Requirement	Priority
Original environmental and data	Soil temperature, light intensity	Medium	Medium
Processed data	Disease and pest feature extraction	Medium	Low
Model parameters and control instructions	Synchronization of irrigation/fertilization decision-making model parameters	Low	Medium
Control data	Agricultural route planning, irrigation and machine start-up instructions	Extremely High	Highest
Emergency and fault data	Extreme weather alerts, equipment fault warnings	High	Highest

**Table 3:** Comparative analysis of key technologies in agricultural edge computing.

Technical Category	Core Advantages	Technical Challenges	Potential Application Scenarios	References
Crop Physiological Monitoring	Real-time, non-destructive interpretation of physiological processes; provides basis for precise nutrition and water regulation.	Deployment and maintenance complexity; challenges in multimodal data fusion.	Precision irrigation scheduling, variable-rate fertilization, early-stage stress alert	[18,19]
Environmental Sensing Technology	Enhances monitoring robustness in complex field environments; on-site preprocessing alleviates bandwidth constraints.	Calibration and synchronization of multi-source heterogeneous sensor data.	Field microclimate monitoring, pest and disease early warning, soil moisture mapping.	[22,23]
Agricultural Product Inspection	n-situ, rapid, and non-destructive inspection; miniaturization.	Complex matrix interference; environmental fluctuations affecting detection stability.	Agricultural product quality grading, rapid food safety screening, origin traceability.	[27,31]
Low-Power Design	Overcomes energy constraints to support long-term maintenance-free operation.	Stability and efficiency of environmental energy harvesting; optimization of dynamic scheduling algorithms.	Long-term monitoring in remote fields, unattended sensor nodes.	[37,38]
Novel Detection Technologies	High sensitivity and non-destructive internal composition analysis; miniaturization and low reagent consumption.	High equipment cost and operational complexity.	Grain/fruit and vegetable quality assessment, on-chip rapid pesticide residue detection.	[42,45]
Model Compression and Acceleration	Overcomes computational and energy constraints of devices to enable efficient inference.	Accuracy-loss trade-off from model compression; hardware platform compatibility.	Large-scale field monitoring, multi-machinery collaboration.	[56,57]
Real-time Decision Engine	High robustness against field uncertainties; millisecond-level response meets high-speed operation requirements.	Difficulties in accurate modeling; complexity in designing adaptive parameter adjustment laws.	Unmanned farm machinery, precise path tracking, coordinated multi-machinery operation scheduling.	[58,60]
Edge–Cloud Collaborative Architecture	Clear division of tasks in a hierarchical architecture; adapts to dynamic and variable field network conditions.	Limited resources of terminal devices; complexity in distributed node management and coordination.	Preliminary sensor data cleaning/feature extraction, real-time machinery control, farm-level resource planning.	[65,70]

**Table 4:** Key technological breakthroughs in the whole-chain traceability of agricultural products.

Application Field	Innovative Technology	Core Mechanism	Application Value	References
Non-destructive Detection	Near-infrared Spectroscopy	Molecular vibration absorption characteristics at specific wavelengths	Reducing raw material supply standardization deviation	[107,108]
Real-time Monitoring	Multispectral Monitoring System	Multispectral image reflection indicators	Improving process controllability	[109,110]
Mycotoxin Detection	Aptamer-based Biosensing	Specific aptamer binding with labeling and signal conversion	Ensuring food safety	[33,111]
Pesticide Residue Analysis	Multi-mode Integrated Detection	Multi-signal fusion and on-site rapid analysis	Reducing pesticide residue risks and ensuring food safety	[112,113]
Field Integrated Platform	Microfluidic Multi-mode Sensing	Multi-mode coordination	Promoting rapid transformation from laboratory to on-site standardized testing	[114]

**Table 5:** Core challenges and impacts of agricultural edge computing.

Bottleneck Dimension	Agricultural Scenario Specificity	Core Impact	References
Communication Reliability	Terrain shielding, dynamic soil dielectric properties, and agricultural machinery vibration	Significant increase in packet loss rate of critical data transmission	[125,129]
Multi-source Vibration Coupling	Mobile platforms, non-uniform terrain	Structural integrity damage to key agricultural components, reduced operational quality	[136,137]
Multi-dimensional Energy Constraints	Energy instability and energy-computing synergy conflicts	Degradation of the reliability of precision agricultural systems	[150,156]
